# Treatment of Urticaria by the Sulphate of Quinine

**Published:** 1851-12

**Authors:** 


*Treatment of Urticaria by the Sulphate of Quinine.*—This is an eruptive disease, usually distinguished by elevations of the cuticle in the form of *wheals;* it is sometimes exceedingly obstinate, resisting all the means that may be brought to bear against it. We are induced to notice this affection, because recently we have met with two or three cases that yielded only to large doses of quinine.

It is often quite simple in its nature, yielding readily to tepid baths, mild cathartics, and a restricted diet; but again, it is accompanied with much febrile disturbance, pain in the epigastrium, nausea, fullness in the head, and a burning sensation over the surface of the body; the face, hands and feet swell; the eyes are almost closed; the tongue is loaded with a white coat, and the itching is intolerable at times. Again, the eruption is accompanied with severe articular pains, all of which phenomena serve to complicate the exanthema, and augment the difficulties of the case. Dr. Wickham and M. Legrouse, of the Hospital Beaujon, report some cases of the worst forms of urticaria, which were promptly cured by full doses of quinine, continued for two or three days.

Treated with quinine the articular pains, the painful tumefaction of the face, feet, and hands, the eruption itself, rapidly disappeared, together with the nausea, febrile excitement, and, indeed, all the distressing symptoms.—*New Orleans Med. and Sur. Jour.*

